# Elaborating Models of eHealth Governance: Qualitative Systematic Review

**DOI:** 10.2196/17214

**Published:** 2020-10-28

**Authors:** Anne Granstrom Ekeland, Line Helen Linstad

**Affiliations:** 1 Norwegian Centre for E-health Research Tromsø Norway

**Keywords:** digital transformations in health care, health policy goals, national and international governance models

## Abstract

**Background:**

Large-scale national eHealth policy programs have gained attention not only for benefits but also for several unintended consequences and failed expectations. Given the complex and mixed accounts of the results, questions have been raised on how large-scale digitalization programs are governed to reach health policy goals of quality improvement and equal access along with necessary digital transformations. In this qualitative systematic review, we investigate the following question: How is governance implemented and considered in the studies included in the qualitative review?

**Objective:**

The aim of this study is to arrive at informed and recognizable conceptualizations and considerations of models of governance connected to eHealth, as presented and discussed in the scientific literature. In turn, we hope our results will help inform the discussion of how to govern such processes to obtain collectively negotiated objectives.

**Methods:**

A qualitative systematic review is a method for integrating or comparing with the findings from qualitative studies. It looks for “themes” or “constructs” that lie in or across individual qualitative studies. This type of review produces a narrative synthesis with thematic analysis and includes interpretive conceptual models. The goal is an interpretation and broadens the understanding of a particular phenomenon. We searched the PubMed database using predefined search terms and selected papers published from 2010 onwards. We specified the criteria for selection and quality assessment.

**Results:**

The search returned 220 papers. We selected 44 abstracts for full-text reading, and 11 papers were included for full-text synthesis. On the basis of the 11 papers, we constructed four governance models to categorize and conceptualize the findings. The models are political governance, normally depicting top-down processes; medical governance, which normally depicts bottom-up processes; the internet and global model, emphasizing international business strategies coupled with the internet; self-governance, which builds upon the development of the internet and Internet of Things, which has paved the way for personal governance and communication of one’s own health data.

**Conclusions:**

Collective negotiations between the nation-state and global policy actors, medical and self-governance actors, and global business and industry actors are essential. Technological affordances represent both positive and negative opportunities concerning the realization of health policy goals, and future studies should scrutinize this dynamic.

## Introduction

### Background

Over the last two decades, large-scale digital health programs and services, such as electronic health records (EHRs), have proliferated with equal access, improved quality, and resource optimization as wider health policy goals. National eHealth policy programs have gained attention not only for their benefits, but also for several unintended consequences and failed expectations. Given the mixed, complex results of these programs, questions are increasingly being asked about the executive power of political-governance strategies and how large-scale digitalization programs are governed to achieve the health policy goals of quality improvement and equal access with the necessary digital transformations.

In this study, based on a qualitative systematic review, we take a closer look at how governance has been implemented and considered in the realm of digitalization in health care by scholars in health care and the health sciences. The objective of the review is to arrive at an informed, recognizable conceptualization and consideration of governance. By summarizing existing and emerging models and the ways they are considered, including suggestions for improvement, we analyze the features of each model and how they possibly interfere with each other. In turn, we hope to inform the discussion on improving governance to obtain eHealth policy goals.

In a 2013 editorial in the *British Medical Journal*, the English national program for information technology (IT) was discussed 10 years after its inception [1]. This program promised to revolutionize care in the English National Health Services (NHS) and was originally scheduled to run for 2 years and 9 months starting in April 2003 [2].

The editorial summarized the results of the national program for IT and discussed its flaws and benefits. We draw attention to the following conclusion on governance: “There have been substantial improvements in the technical knowledge base underpinning information systems in the NHS, in organizational capacity to introduce any new IT system, and in information governance processes and procedures. Ten years on, only a handful of hospitals can be described as paperless, and most communication between NHS organizations still occurs by snail mail, fax, or patient messenger” [1].

Governmental opponents of the program described it as a huge disaster, but in the final benefits statement released by the Department of Health, its proponents largely described it as a success. They predicted that by 2022, financial benefits would be £10.69 bn (US $13.86 bn), outweighing the cost of £9.78 bn (US $10.09 bn).

Similar accounts of the contested, intended, and unintended results of eHealth programs were described in the United States in 2019: “The US health system has recently achieved widespread adoption of EHR systems, primarily driven by financial incentives provided by the Meaningful Use (MU) program” [3]. Although successful in promoting EHR adoption and use, this program and other contributing factors also produced important unintended consequences, such as failed expectations, EHR market saturation, innovation vacuum, physician burnout, and data obfuscation, with far-reaching implications for the United States health system [4]. To avoid unintended consequences, these authors proposed improved governance, including efforts from diverse players such as health care providers, administrators, health information technology (HIT) vendors, policymakers, informatics researchers, funding agencies, and outside developers. The authors also argued for the promotion of new business models, collaboration between academic medical centers and informatics research departments, and improved methods for evaluating HIT.

These accounts of mixed, complex results, challenges to the realistic calculation of benefits and costs, and implementations of intentional changes constitute the background of this paper. Perhaps unintended consequences could have been minimized through stronger, better governance of these processes in England and the United States.

At the outset, we understand health care governance as a political process regarding national eHealth goals. National health care services differ between countries and regions, and they are broadly categorized into 2 systems: single-payer and multi-payer systems [5]. National authorities are responsible for governing their respective national health care services, including eHealth governance strategies that are often top-down through legal regulation and reimbursement schemes, where resources are allocated to prioritized service providers. However, political governance can be driven by evidence-based medical imperatives, which are bottom-up strategies [6]. National eHealth policy adjustment in such cases occurs according to new medical evidence.

Governance is extensively discussed in social and political sciences and is one of the most common social science terms [6]. One prominent definition is “an interactive process through which society and the economy are steered toward collectively negotiated objectives” [6]. Political goals are normally the result of compromises obtained through collective negotiations between stakeholders. “Public governance should thus be considered as composite and mixed with inbuilt tensions between competing concerns, actors, ethics, resources, and time horizons” [6,7].

This paper considers eHealth governance as a dynamic process in which development, decisions, implementation, evaluation, and adjustments overlap and interact. The development grows out of conscious and unconscious interdependencies. To realize deliberate policies in such interdependent situations, collective negotiations might be increasingly important. In Norway, the word “co-management” captures this process, which blurs the strict distinction between bottom-up and top-down governance.

One group of researchers has emphasized the need for more empirical studies in the field of governance to strengthen both conceptual and empirical knowledge [8].

We report on the results of a systematic, qualitative review of how eHealth governance has been conducted and experienced. We investigate how governance has been addressed and has played out in eHealth programs as described in the health sciences literature. We do not aspire to compare different enactments of governance, but instead, perform a descriptive analysis by conceptualizing the characteristics of governance and the experiences presented in the cited papers. We also discuss how different models interfere with each other and summarize suggestions for improvements.

We have restricted our search to PubMed because digital health is a popular emerging topic, and it is of interest to learn how governance is addressed and considered in health sciences. On the basis of political and social sciences, we contribute to a cross-fertilization of scientific traditions by communicating with practitioners, medical internet researchers, and political health authorities.

### Question

How do the papers included in the qualitative review define and consider governance?

### Objectives

The objective is to arrive at informed, recognizable conceptualizations and considerations of governance models of eHealth, as presented and discussed in the scientific literature. In turn, we hope our results will help inform the discussion on how to govern such processes to obtain collectively negotiated objectives.

### Outline

The remainder of the paper contains an account of the methods used, quantitative and qualitative results, and a discussion section in which we distinguish between and elaborate on models based on our constructs from interpreting the empirical results of the cited papers. We discuss suggestions for governance improvements, present our conclusions, and point to areas for further research. We also discuss the limitations of this review. Due to the coronavirus pandemic, we have provided a postscript that connects our models and conclusions to possible (eHealth) governance in the COVID-19 era.

## Methods

### Qualitative Systematic Review

A qualitative systematic review is a method for integrating or comparing with the findings from qualitative studies. It looks for themes or constructs that lie in or across individual qualitative studies and may employ selective or purposive sampling. This type of review produces a narrative synthesis with a thematic analysis and includes interpretive, conceptual models. The accumulated knowledge resulting from this process may lead to the development of a new theory, an overarching narrative, a wider generalization, or an interpretative translation. The goal is not aggregative in the sense of adding studies together, as with a meta-analysis; rather, it is interpretative and broadens the understanding of a particular phenomenon [9].

### Search Strategy and Information Sources

Our search was performed in November 2018 using the previous version of PubMed. PubMed was updated in the spring of 2020.

We searched “All fields” with the following search criteria:

Search (“governing reforms” OR (telemedicine governance) OR (governance “national telemedicine programme”) OR (governance “national ehealth programme”) OR (governance “national ehealth programme”) OR (governance innovation ICT) OR (“innovative procurement” health*) OR (“innovative procurement” health) OR (“whole system demonstrator programme” lessons) OR (Sundhedsplatformen) OR (Governance “regional EHR implementation” Denmark)).

The search was restricted to scientific papers, including systematic reviews published in peer-reviewed journals. A systematic review was defined as an overview with an explicit question and a methods section with a clear description of the search strategy and methods used to produce the review. The review was also expected to report on and analyze empirical data. As many papers were retrieved (220), we included only reviews and papers published from 2010 onwards in the final review for pragmatic reasons.

### Inclusion Criteria

#### Population and Participants

We included papers on the governance of national, regional, or global eHealth programs focusing on population, public health, hospitals, communities, patients, consumers, health professionals, and family caregivers, regardless of diagnoses or conditions.

#### Interventions and Issues

The review included governance issues connected to all eHealth interventions, information and communication technologies (ICT) for communication in health care, and internet-based interventions for diagnosis, self-management, and treatments. Social care was considered relevant if it was an important part of health care and occurred in collaboration with health care for patients with chronic conditions.

#### Comparisons

Papers that compared governance of eHealth and governance of standard care or other types of care were included in the review.

#### Outcomes

Only papers and reviews that reported relevant outcomes were included. Relevant outcomes were specified as eHealth governance with health-related outcomes (morbidity, mortality, quality of life, and patient satisfaction), process outcomes (quality of care, professional practice, adherence to recommended practice, professional satisfaction, governance strategies, organizational aspects, policy and implementation), and costs or resource use. Systematic reviews and papers reporting on emerging issues, such as unexpected findings or important new insights, were also included.

#### Languages

Articles published in English, French, or a Scandinavian language were included.

### Exclusion Criteria

The governance of general health reforms, innovation programs, public reforms, and innovation programs was excluded, as were conference papers, dissertations, proceedings, and irretrievable papers.

#### Design

Papers and reviews considered nonsystematic or nonrigorous, such as commentaries, editorials, and proceedings, were excluded, as were systematic reviews with major limitations (low quality) according to a revised checklist for systematic reviews from the Cochrane Effective Practice and Organisation of Care Group. If the same authors had produced several publications of the same review, the most updated and/or the fullest review was selected while other versions were excluded.

#### Participants

Studies with participants considered irrelevant to the review were excluded, such as studies on the use of ICT outside the health care domain. Animal studies were also excluded.

#### Interventions Considered Irrelevant to the Review

Studies on the governance of interventions considered irrelevant to the review included internet-based education of students and health professionals, medical technology in clinical practice in general, such as medical and surgical examinations and treatments based on computer technologies, except when used in remote diagnosis and treatment (telehealth); the use of telephones (including cell phones) only; eHealth as only a limited part of an intervention; and the use of the internet for surveys, research, web-based prescriptions, mass-media interventions, and veterinary medicine.

#### Outcomes

Articles without relevant outcomes were excluded, that is, those not meeting the inclusion criteria.

### Study Selection

Articles were stored in the free software Rayyan, where we inserted the selection criteria and read the abstracts [10]. On the basis of the inclusion and exclusion criteria, two reviewers independently screened the list of the titles and abstracts from the literature searches and identified potentially relevant studies, the full-texts of which were then retrieved. We resolved any disagreement through dialogue between the two reviewers based on the selection criteria. The citations were then exported to EndNote.

### Data-Collection Process

Data collection was carried out using a web-based data-extraction form created by the authors in Google Docs, which is enclosed in Appendix 1. The 2 authors collected data based on full-text papers. The following quality domains were assessed to identify, include, and critically appraise the studies: explicit accounts of data-collection methods, explicit accounts of methods used to analyze the findings, and overall assessments of the qualities of the papers and reviews.

### Analytical Perspective

We analyzed the papers from a grounded perspective by interpreting the qualitative data in the included papers. Grounded theory is a form of empiricism that emphasizes inductive reasoning and hypothesis generation, in contrast to the hypothetic co-deductive model of the positivist scientific method [11]. Our interpreted models can be used as working hypotheses for further empirical development and research.

## Results

In total, we included 44 of the 220 abstracts extracted from PubMed.

### Quantitative Results

**Figure 1:**
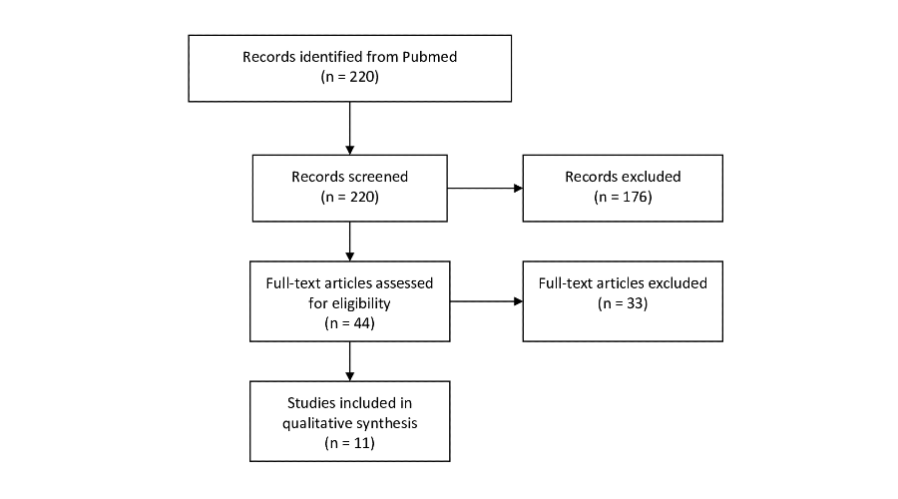
PRISMA (Preferred Reporting Items for Systematic Reviews and Meta-Analyses) flowchart.

The Preferred Reporting Items for Systematic Reviews and Meta-Analyses diagram below (Figure 1) presents the quantitative results [12].

### Qualitative Results: Governance Models

In the background section, we briefly indicated 2 broad governance strategies: political governance, which normally depicts top-down processes and medical governance, which normally depicts bottom-up processes. On reviewing the 11 papers, we found it difficult to subsume all of them under either political or medical strategies.

Therefore, we constructed four governance models to categorize and conceptualize our findings. We chose to construct models because “Models seek to simplify phenomena as an aid to conceptualisation and explanation” [13]. They are also tools to realize a narrative synthesis, which includes thematic analysis and interpretive, conceptual models.

The 4 models are: political governance, medical governance, internet and global business governance, and self-governance. We briefly describe the models below before sorting the different papers into them.

1. Political governance: Health policy and implementation are nation-state responsibilities. However, nation-states are also members of the World Health Organization, which has a global perspective on governance. Regional or local authorities are also responsible for collectively negotiating goals. National authorities can choose top-down governance approaches or delegate responsibility to the regional and local levels to facilitate bottom-up strategies. Some papers described this up-and-down process as a national “middle-out” approach to eHealth decision-making. We have conceptualized all these strategies as political strategies, implying that the top-down perspective is broad and dynamic, as our background section indicates.
2. Medical governance: The medical model implies that governance initiatives and executive agencies rest with medical and health care professionals and professional organizations, which provide the terms of development. This model also comes with dynamic adaptations, such as adjustments to the political model. In radiology, for instance, which was fully digitalized at an early stage, radiologists saw its national medical potential and consequently influenced political strategies.
3. Internet and global business governance: In this category, we include governance mechanisms by nongovernmental organizations, the health care industry, and private internet companies. The structure of the internet facilitates new business models, which may challenge political strategies and national control. The internet is accessible to almost everyone, and no international quality assurer exists, which prohibits the diffusion of information or services that might contradict medical evidence and challenge public health. Illicit drug sales on the internet is an example of a new business model.
4. Self-governance: This model builds on the development of the internet and the Internet of Things (IoT), which has paved the way for the personal governance of health by storing, developing, and communicating one’s own health data. This model considers eHHealth to be developed by individuals’ efforts to optimize their own health. The self-management of diabetes through apps, for instance, may facilitate individual governance (by patients and citizens) of eHealth services through demand for new apps and simultaneously empower patients and strengthen their digital literacy.

### The Political-Governance Model

The following 4 papers align with the political-governance model: Atalag addresses a single content model for eHealth interoperability and secondary use in New Zealand [14]; Park and Atalag address the national approach to health care ICT standardization and focus on progress in New Zealand [15]; Kierkegaard addresses interoperability after EHR deployment and describes persistent challenges and how the Danish government applied a combination of bottom-up and top-down strategies to realize full-scale EHR implementation [16]; de Riel et al [17] address success factors for implementing and sustaining a mature electronic medical record, iSante, in a low-resource setting in Haiti (Table 1).

In the political-governance model, eHealth is governed top-down through a national eHealth strategy. The papers included in this model describe national top-down strategies on technical infrastructure, legal frameworks, and institutional structures, such as national advisory boards or a multi-editorial expert group [15,16] (Table 1).

Atalag describes the national top-down strategy of New Zealand as a “middle-out transitional approach to achieving semantic interoperability in eHealth” [14]. Kierkegaard describes how the Danish authorities combined top-down and bottom-up approaches to realize national eHealth goals: “Changes in the organizational setup and redistribution of responsibilities between the Danish regions and the state play a pivotal role in producing viable and coherent solutions in a timely manner” [16].

**Table 1:** Included papers, governance models, and strategies.

Reference	Country	1: Political governance	2: Medical governance	3: Global internet governance	4: Self-governance
Atalag K, 2013 [14]	New Zealand	Single content model; top-down strategy – framework for standards	N/A^a^	N/A	N/A
Park YT, 2015 [15]	New Zealand	National Health Board and National Health IT Board; top-down strategy	N/A	N/A	N/A
De Riel E, 2018 [17]	Haiti	The Haitian Ministry of Health Management Unit; top-down strategy for a national electronic medical record model	N/A	N/A	N/A
Kierkegaard P, 2015 [16]	Denmark	The National eHealth Authority; top-down strategy – frameworks for standards	N/A	N/A	N/A
Wade, VA 2012 [18]	Australia	Governmental rebates for medical specialists using telehealth No general IT for health top-down strategy	Telehealth as a game-changer in clinical practice in ethical, legal and medical governance aspects	N/A	N/A
Crocker M, 2010 [19]	United Kingdom	N/A	Neuroscience centers organized as a clinically driven tertiary referral service; bottom-up strategy from the medical society (radiology)	N/A	N/A
Sutton LN, 2011 [20]	United Kingdom	N/A	National PACS^b^ part of the National Program for IT after reacting to bottom-up strategy on realizing benefits	N/A	N/A
Bagot KL, 2017 [21]	Australia and United Kingdom	N/A	Creating sustainable medical networks in stroke care; cultural differences in medical governance; bottom-up strategy	N/A	N/A
Mackey TK, 2014 (A call for a moratorium) [22]	Global	N/A	N/A	Internet Corporation for Assigned Names and Numbers, nonprofit organization; bidding process and dot-health (health)-industry business strategy	N/A
Mackey TK, 2014 [23]	Global	N/A	N/A	Internet Corporation for Assigned Names and Numbers chose the International Chamber of Commerce to adjudicate dot-health concerns	N/A
Williams SJ, 2015 [24]	Global	N/A	N/A	N/A	Self-management and web-based community member outside national health services; self-health-optimizing strategy

^a^N/A: not applicable.

^b^PACS: Picture Archiving and Communication System

### The Medical-Governance Model

The following 4 papers align with this model: Wade et al [18] presented a qualitative study of ethical, medico-legal, and clinical governance matters in Australian telehealth services; Sutton considers a Picture Archive Communication System and diagnostic imaging service delivery from the UK perspective [20]; Bagot et al [21] performed a qualitative analysis comparing with the experience of Australia and the United Kingdom in integrating acute stroke telemedicine consultations into specialists’ “usual practice”; and Crocker et al [19] address patient safety and image transfer between referring hospitals and neuroscience centers, exploring possible methods of improvement.

The bottom-up strategy may grow out of local needs [18,20], and sometimes it is picked up and transformed into a national top-down strategy. For instance, Crocker et al [19] claim that “Part of the remit of the National Programme for Information Technology in the NHS (NPfIT) was to improve neuroscience teleradiology.” The researchers wanted to evaluate whether their experiences were part of the national top-down strategy; so the paper describes how the medical society experienced their recommendations presented in an NHS report from 2004 being “largely ignored” in the national program. National top-down strategies do not necessarily align with bottom-up strategies [19].

Wade et al [18] state that, from a medical point of view (bottom-up), concerns about data security in telemedicine need to be governed through national top-down guidelines.

### The Internet and Global Business–Governance Model

The following 2 papers align with this model: Mackey et al [23] addressed health domains for sale and argued for the need for global political health governance on the internet, and Mackey et al [23] discussed a call for a moratorium on the top-level domain “generic health” to prevent the commercialization and exclusive control of web-based health information [22].

These authors argue that the national jurisdiction of privacy and data protection is largely ignored and challenges existing international governmental organizations, such as the World Health Organization and European Union [7]. According to the authors, the business interests of large actors and market mechanisms overrule both political and medical governance.

Mackey et al [22] claim that the processes of the Internet Corporation for Assigned Names and Numbers (ICANN) “appear to favour business interests and generation of profits over the future integrity of the Health Internet, failing to make any tangible commitments to protect public health or enforce norms as would be found in a responsible global governance Framework.”

However, they also state that “Direct-to-consumer advertising of prescription products is not allowed in the vast majority of countries other than the United States and New Zealand, and it may be unlawful for pharmaceutical manufacturers to engage in multijurisdictional web-based advertising that could occur through future gTLDs (generic top-level domain name)” [23]. There is a lack of global governance in health that may challenge human rights in public health [23].

### The Self-Governance Model

We have included one paper in this category by Williams et al [24], who discussed governance in terms of individual agency as opposed to political governance. They also envision the opposite of individual agency: the ways in which digitalization and the use of technological affordances change individuals and identities. The paper describes a complex model, a dynamic dualism between the governance of individuals by eHealth versus the self-governance of eHealth.

We have presented the included papers under 4 governance models: political governance, which is normally limited to nation-state jurisdiction, international, evidence-based medicine and health care guidelines, large-scale industries governed by business goals, and self-governance by consumers.

### Summary of Empirical Results

Table 1 lists the 11 papers by author, year published, country in which the governance model is discussed, and model or strategy. Some papers do not refer to a specific country because they describe global models or models related to the consumers’ use of the IoT products and services.

Table 2 lists the included papers by author, year published, country in which the characteristics, experiences and challenges are described, and the suggested improvements related to a specific governance strategy. Three papers do not relate to a specific country but describe aspects related to global governance and self-governance models.

**Table 2:** Included papers, considerations, and emerging governance trends.

Reference	Country	Experience and performance (descriptive)	Considerations and suggested improvements (descriptive)	Trends and emerging governance models
Atalag K, 2013 [14]	New Zealand	Suggesting a “middle out” approach; a single top-down approach does not produce the expected outcome; success relies on a relationship based on trust between the authorities and the medical society	The national Single Content Model is flexible and “enables smooth transition to a comprehensive solution through gradual replacement over time”	The openEHR trend and Archetypes; governance based upon a top-down strategy for interoperability
Park YT, 2015 [15]	New Zealand	Strong top-down governance strategy and organizational structures and rules has been the most important factors for successful eHealth governance	Next steps will be to analyze how the eHealth structure influence health outcomes and minimize errors	Strong belief in strong government involvement leads to successful goal attainment in eHealth
De Riel E, 2018 [17]	Haiti	A key learning is leadership engagement to create an understanding of how the system works	Haiti’s health care service is dependent on national and third-party funding; realizing a sustainable information and technology communications infrastructure is a muddle-through process	System migration to the OpenMRS platform to take advantage of a global community and ensure sustainability
Kierkegaard P, 2015 [16]	Denmark	Setting national goals and adapting the middle-out approach as part of a national strategy is the best way to realize full-scale implementation of electronic health records, an approach based on cross-regional coordination	A collective phase-out of all systems may be costly, but it may be the only way to create a common national platform of high interoperability; risk of regions working with one vendor, which creates regional dependency on an actor with market monopoly	The Danish framework is flexible and in line with the European Union eHealth Interoperability framework
Wade VA, 2012 [18]	Australia	This study indicates that telehealth can be a tool to realize medical quality of care governed bottom-up and in line with evidence-based medicine	The medico-legal aspects did not seem to be as difficult as anticipated; national reimbursement schemes may increase substantial system benefits	N/A^a^
Crocker M, 2010 [19]	United Kingdom	Image transfer is delayed by an immature technology infrastructure	Bottom-up recommendations from the teleradiology society in United Kingdom has been ignored by the top-down process	N/A
Sutton LN, 2011 [20]	United Kingdom	Creation of a UK reporting “Grid” with remaining organizational and governance challenges	Telehealth challenges around “team organisation” when the reporter and doctor are in separate organizations at a distance	Creation of a medical governance model in radiology across the United Kingdom which harmonize with the national top-down strategy of National Health Services
Bagot KL, 2017 [21]	Australia and United Kingdom	UK model: telemedicine integrated in the specialist work plan; Australia telemedicine was ad hoc	Both networks see telemedicine as part of future organization; can reduce workload by a “follow-the-sun model”	N/A
Mackey TK, 2014: (A call for a mora-torium) [22]	Global	The governance of health-related internet domains should be run by international organizations and not by a for-profit company like Internet Corporation for Assigned Names and Numbers, which does not respond to actors who want this policy change	The international society should stop the Internet Corporation for Assigned Names and Numbers governance model	Is it feasible to realize a global internet governance model based on international interstate cooperation?
Mackey TK, 2014 [23]	Global	The governance model of dot-health and other internet health domains is not run by the World Health Organization or another international health organization, which may represent a global threat to public health: access to evidence-based medicine and quality assured health information	The main challenge is to realize a shift from a privately-run model to an international model governed by legitimate international public health actors	The combination of internet and global markets challenges political and medical governance
Williams, SJ, 2015 [24]	Global	Health apps are a growing trend that realizes “the quantified self”: blurring the lines between health care and wellness through data sharing	N/A	“Quantified-self” apps contribute to a growth of health and wellness data; a “global health and wellness data governance model”?

^a^N/A: not applicable.

## Discussion

### Elaborating Models, Experiences and Suggestions for Improvement

This section focuses on the features and considerations of the different models, the ways in which they interfere with each other, and suggestions for improved governance.

### Features and Considerations of the Different Models

#### Features and Considerations of Political Governance

The 4 papers in this category describe top-down governance models and discuss how top-down national policy has used regional eHealth models as tools to implement wider national health strategies through a defined framework and governmentally assigned top-level working groups.

When, for instance, the Danish government wanted to speed up EHR implementation, they focused on regional governance to replace their former top-down strategies. Likewise, when the government found it more suitable to decrease the number of existing EHR systems, they strengthened the top-down national model by using regional middle-out strategies [16]. The government encouraged cross-regional coordination as part of the national eHealth strategy (Table 2).

The history of Denmark’s health care system demonstrates the inherent difficulties of a state-centric approach to technology harmonization. Similar results have been obtained when other countries applied a top-down approach to national-level shared medical records, such as the ambiguities in the NHS National Programme for IT (NPfIT). Moreover, Denmark’s methods for overcoming the interoperability issues between EHRs demonstrated that health information exchange is not merely a technical issue, “but a challenge fraught with organisational and political complexities” [16].

In New Zealand, the government governs the top-down implementation of eHealth using a national eHealth strategy and standards. Atalag argued that it was difficult to achieve a high level of interoperability through a top-down strategy, partly because of resistance from different organizational actors. The model was therefore replaced by “a middle-out (transitional) approach to achieve semantic interoperability in eHealth” [14]. This approach was strategically used temporarily until the top-down approach was resumed (Table 2).

The Haitian model included three multi-site electronic medical records as the cornerstone of the broader planned national eHealth architecture, eventually feeding into an overarching system to aggregate health-indicator reporting (called Syste`me d’Information Sanitaire National Unique). The Haitian Ministry of Health set this model as a priority in 2013 [17], a middle-out approach that included both the top-down governmental strategy, the bottom-up medical strategy, and the donor strategy as a third actor. This middle-out approach helped realize health policy goals related to sustainable health service.

The case from Haiti shows that a top-down strategy may apply in low-resource, donor-dependent settings. “Strong leadership was essential to system continuity and expansion. In addition, many developing countries may not have a legal framework that addresses management or protection of health information” (Table 2).

In New Zealand, Atalag emphasized that “the success of the described approach relies heavily on appropriate governance, and it is imperative to put in place new models of collaboration” [14]. The government of New Zealand, unlike those of other countries, established specific HIT bodies to drive and support standardization, including the National Health IT Board and the Health Sector Architects Group, which actively participated in the standardization processes (Table 1). They collected ideas from the health care industry and the public sector and developed HIT standards. In the second paper, Park and Atalag further emphasize that New Zealand’s governmental structures and processes may be a direct and efficient way of achieving the benefits of HIT standardization because government power has a strong inﬂuence on markets, and many health care organizations are under government control [15].

In summary, political-governance models encompass bottom-up, top-down, and middle-out approaches. We chose to label them top-down however, because even bottom-up strategies are a part of top-down governance since health care is a nation-state responsibility. These strategies seem to fluctuate dynamically within the nation-state, following a thesis-and-antithesis curve. Bottom-up and middle-out strategies seem to be shifting to improve top-down governance incrementally and instrumentally. The models enact governance *through* and *of* eHealth: influential human actors use eHealth programs as tools, and shifting strategies are applied to obtain wider health political goals.

#### Features and Considerations of Medical Governance

The political-governance model’s top-down focus on governance by standards, legal frameworks, and national boards may lead to an underestimation of other important aspects affecting governance, such as knowledge, identity, roles, cultures, trust, quality, and asymmetric power relations.

Medical governance may shed light on these aspects of governance in eHealth. Medical governance is enacted within medical networks by medical and health care professionals, who see potential in technology and create networks to maximize its benefits and improve medical practice. “Clinical governance is a systematic approach to implementing quality and safety in health care, which aims to maximize evidence-based practice and reduce risk” [18]. In this paper, Wade et al [18] suggest that various examples have shown that telemedicine can be used to enforce medical governance as a driving force to support the uptake of evidence-based care because it has been difficult to change organizational cultures in health care systems. The literature on the organizational effects of telehealth has been sparse and has focused on the details of implementation. Wade et al [18] further comment on governance within eHealth communities: “Telehealth could be a key factor in quality improvement as it produces immediate contact between providers, is based on the management of real patients and promotes trust in interprofessional relationships” (Table 2).

Bagot et al [21] argue that clinicians must adapt to this new way of delivering services; adaptation subsequently affects the trust and roles and responsibilities between organizations that collaborate on eHealth governance: “Successful telemedicine networks require specialists adapting clinical practice to provide remote consultations.” The authors indicate a knowledge gap with respect to medical governance between different countries (Table 2).

Sutton discusses experiences in medical governance as part of the development of telemedicine networks: “It is important for local health care communities and their patients to ensure teleradiology does not destabilize or de-skill smaller departments. Teleradiology should be complementary and not an alternative to progressing development of the service locally by enhancing the expertise of the local radiology workforce” [20].

Radiology in England has undergone reorganization due to digitalization. Currently, following the merger of clusters into three main areas across England, there are three major Picture Archiving and Communication System providers with associated storage archives [20]. This process includes the medical society bottom-up: “The Radiology Service Improvement Team of the NHS . . . is now part of the NHS Institute for Innovation and Improvement” [20] (Table 2).

Other authors also note that the medical society opined that the teleradiology recommendations of the 2004 NHS Modernization Agency Neuroscience Critical Care Report have been largely ignored, even though “part of the remit of the National Program for Information Technology in the NHS (NPfIT) was to improve neuroscience teleradiology” [19].

The included papers demonstrate that implementing telemedicine services requires inter- and intraorganizational cooperation and raises questions that transcend standards, infrastructures, and legal frameworks. These include implementation challenges, clinicians’ trust, and the associated uncertainty around how best to ensure stable access to skilled personnel at local departments. There is also uncertainty about clinical accountability and responsibility. Clear governance of these aspects is considered crucial to clinicians.

Including high-ranking medical professionals in the implementation process of national programs, such as the NPfIT, is suggested to increase clinicians’ trust in national implementation. In cases where the medical society considered their input largely ignored, trust was lost in the top-down strategy. However, these papers do not describe how the implementation process is affected by the lack of trust from the medical society.

Other scholars who have evaluated national EHR implementation programs point out similar results: Strategic, organizational, and human challenges are usually more difficult to master than technical aspects” [25].

In summary, the medical model addresses governance within and by professional eHealth communities. Medical governance represents the bottom-up and middle-out perspectives. The papers describe how medical governance systematically outlines how digitalization maximizes evidence-based practice, reduces risk, and thereby increases quality and access to care. Trust, responsibility, role definition, and skill development are considered crucial to obtaining evidence-based results. This model also faces ongoing challenges that require serious effort to succeed.

#### Features and Considerations of Internet and Global Business Governance

In the governance literature, the term “organized anarchy” is used to describe a “loosely coupled” organized structure, which refers to a “relatively open and unspecialized” structure [7]. We interpret this model to denote the internet as a technology with affordances that facilitate organized anarchy. It transcends the jurisdictions and economies of the nation-state and allows global discovery and innovation. Technology facilitates the realization of this anarchy, opening the possibilities for actors to create organizations and services beyond national jurisdiction. eHealth can be part of this global development.

The 2 papers included describe how the internet, which consists of a hierarchical domain-naming system for the internet protocol addresses of computers, services, and other digital resources, relies on domain names as an easily recognizable way for users to search and navigate web-based content. “The Internet Corporation for Assigned Names and Numbers (ICANN), a nonprofit corporation founded in 1998 that controls this system, is currently undergoing the largest expansion of the internet in history” [23].

ICANN is a nongovernmental organization established by the United States government but is notionally independent of it. It manages this hierarchical naming system and roughly 500 accredited domain-name registrars. “ICANN relies on an international Board of Directors consisting of various ICANN constituents, a CEO, staff, and advisory committees consisting of stakeholders from national governments, Internet technical experts, and Internet organizations to inform its decisions” [22].

The authors of the two papers are critical of the ways ICANN governs, noting that it has created “the largest expansion of the Internet in history . . . adding over a thousand new generic top-level domain names (gTLD), potentially including a new .health domain and close to 20 other gTLDs related to medicine and health” [23]. They consider that ICANN’s complex, highly political process of awarding health-related gTLDs profoundly impacts information privacy, use, and sale as well as health marketing and content quality, which could influence future trust, security, and credibility of the Health Internet. Hence, they argue that it is critical that applicants are carefully scrutinized to ensure that they are abiding by ethical principles, practices, and rules with respect to public health and the public interest.

More than 100,000 health-related websites are estimated to exist, and internet users may have difficulty accessing evidence-based sources and often seek information through simple search-engine queries (eg, Google, Yahoo, and Bing) that may prioritize sites of lower quality, undisclosed commercially sponsored content, irrelevant information, and/or misinformation. “For example, illicit online pharmacies have been detected illegally marketing and selling pharmaceuticals without prescriptions, misrepresenting crucial risk information, and not disclosing other risks of their often counterfeit and otherwise dangerous products” [23]. “Because it governs the majority of the domain name system, ICANN bears great responsibility for those standards, and how the Internet can be used to help or harm individual users” [22].

Objections to ICANN’s governance model have come from the World Health Organization, scholars, and international public health organizations. In 2000, the World Health Organization and other stakeholders proposed the formation of health top-level domain names (TLD), but ultimately their proposal was not chosen as one of the seven proof-of-concept names for new TLDs in that round [23]. The authors underline that ICANN lacks “enforceability, because it has no appeal process to take proactive action against websites that violate laws that accredited registrars fail to report. Consequently, many websites feature illicit online content with clear public health and patient safety concerns that registrars take no action against, such as websites selling medicines without a prescription and that also potentially traffic counterfeit or falsified medicines” [22].

The authors give reasons to worry that the situation will be exacerbated by simply awarding new health domains to the highest bidders. “ICANN’s processes appear to favor business interests and generation of profits over the future integrity of the Health Internet, failing to make any tangible commitments to protect public health or enforce norms that would be found in a responsible global governance framework” [22].

According to the United Nations, health is a fundamental human right. More people than ever are using the Health Internet to seek information and make behavioral choices. “Now is not the time either to compromise this legal right or complicate the factual reality, in favor of profit-making interests merely for the sake of unlimited Internet expansion” [22].

The authors argue that governing health-related internet domains should be a priority for international public health organizations as well as global IT organizations, such as ICANN, the World Health Organization, the International Telecommunication Union, the World Summit on the Information Society and its Internet Governance Forum. ICANN, as a nongovernment organization with a global autonomous governance structure, does not seem to make any tangible commitments to protect public health or enforce norms, as would be found in a responsible global governance framework [22].

In summary, this model exceeds the political and medical governance models and puts international business actors in a dominant role. The authors envision a rather dystopian future, especially for the governance of health data and services on the internet, and they argue for action by global health authorities.

#### Features and Considerations of Self-Governance Models

We have included one paper in this category. It describes how sleep-monitoring and diagnosing sleep problems and treatment were moved from medical governance to a self-governance model. This “health service” takes place outside health care institutions and is facilitated by the IoT, which may exist at the global level beyond nation-state jurisdictions. In this model, nongovernmental or private actors may deliver services outside the national health system, as discussed in the previous chapter.

The difference between these devices and previous portable devices is that the user–technology relationships configured by new digital sleep-monitoring technologies are primarily between sleepers and devices. “The information about sleep feeds directly back to the user, providing sleepers with new knowledge about their dormant (or not-so-dormant) body/self; knowledge that itself is imbued with a sense of responsibility for them to act to improve their sleep” [24]. The authors argue that sleep, or the sleeping body or self, is yet another site for improvement or optimization in terms of performance, and that health is important well beyond the clinical sphere.

Further, they point out a certain seductive power in tracking, monitoring, and managing ourselves in the interest of self-knowledge, or even self-governance, for self-improvement or self-optimization.

Referring to Deleuze, the authors warn against forms of more or less continuous control [23,26]. Further, they envision submission and postpanoptical surveillance, referring to Massumi, Foucault, and Bauman [27-29].

In summary, self-governance enacts a dynamic between global internet governance that forces or nudges humans, on the one hand, to adhere to technologies and connected health businesses, and on the other, to rely on them as sources of individual self-governance and control. Governance is characterized by a shifting, dynamic tension: governance of the individual by eHealth (submission) versus self-governance within eHealth services, involving empowered, conscious citizens.

#### The Ways the Models Interfere With Each Other

Wade et al [18] argue that medical governance needs help from political governance to regulate responsibility, ethics, and accountability by stating that it is unclear whether responsibility should rest with the primary clinician or be divided between the local clinician, the distant clinician, and the technology provider. They substantiate this by asserting that this responsibility is not regulated in Australian law, noting that there is “no case law relevant to telehealth in Australia” [18]. In addition, they expect this to change: “These matters have not been resolved in Australia, although with the advent of universal telehealth rebates, standards for practice are under development by the professional colleges” [18].

Top-down political-governance strategies are used by national authorities to ensure national frameworks of standards and legal aspects, and medical governance seems to be used as a bottom-up model and strategy to ensure that the process is anchored and incorporated into medical practice. In the cases in Denmark and New Zealand (Tables 1 and 2), both governance models were expected to work in tandem. When the tension between them grew or the governments became aware of possible tensions that could slow the process, they used a middle-out approach to increase the legitimacy of the top-down eHealth strategy.

The middle-out approach is often defined by national advisory boards, which include multiple stakeholders. This was the case for Denmark, New Zealand, and Haiti, which recognized tensions between stakeholders and consequently included medical professionals, vendors, patients, and bureaucrats in their eHealth governance models to facilitate and enforce negotiations. eHealth governance is recognized as an arena for collective negotiations to co-manage tensions to realize national health policy goals.

The global internet and business model describes challenges concerning forces that are complex, ubiquitous, unpredictable, and ungovernable by established policy structures and strategies. This model defines governance in terms of challenges in the governance of health-related data and digital services. Such challenges occur because of interactions between technological affordances and international business actors.

According to the 2 papers on ICANN, the internet and global model interferes with the other 3 models because the ICANN overrules World Health Organization efforts.

The political models were based on the abilities of nation-states to regulate and govern eHealth policy alone or through international cooperation in international governmental organizations or supranational organizations. The anarchical structure of the internet adds a challenge to the political and medical governance of eHealth; however, combined with the internet’s global outreach, it facilitates business growth and reduces political and medical governance, leaving it up to the consumer to validate services and products. The European Union passed the General Data Protection Regulation to precisely address this challenge and protect consumers [30].

Self-governance may fit into wider patterns of voluntary and involuntary submission, particularly in the digital era of big data, which Williams et al [24] call “creeping forms of monitoring and surveillance that seem to characterize our lives today within and beyond the medical and health domains.”

Such submissions may also be used by global web-based businesses to target services to specific user groups and control markets. Thus, there is a tension between self-governance and global internet and business governance concerning control and manipulation.

### Elaborating Suggestions for Governance Improvements

Scholars in the political-governance category articulate the need for more documentation of the successful, locally-led governance of donor-funded systems, including any capacity-building for local responsible entities and joint system design, planning, and implementation [17]. Experiences with political strategies point to a demand for ongoing efforts to improve them, with stronger political executive power building on medical relevance to achieve wider goals.

In the medical-governance category, scholars point out the need for continued monitoring of a more robust image-routing system across the NHS and audits of its functionality by the Connecting for Health Safety Team and the National Patient Safety Authority to combine bottom-up and top-down strategies [19].

In support of this argument, Sutton claims that “There is much hope that despite the current economic climate, the established IT programmes in the UK are able to continue to facilitate the development of new solutions. In 2010, the Department of Health in England adopted the ‘Quality, Innovation, Productivity and Protection’ agenda to maximize the benefits of existing IT systems and promote improved patient care and productivity” [20].

In a paper concerning ethical and legal matters in Australian and UK telehealth, the authors reported that Conducting organizational case studies would give a deeper understanding of the matters identified, particularly those of governance and system change” [18]. In their comparison of the integration of acute stroke consultations and specialists’ usual practice, the authors suggest that “Future research might investigate the transferability of UK and Australian experiences to broader European, Asian and American networks” [21]. In general, medical culture is similar across national borders.

The medical society is global and agrees on evidence-based medicine being the core driver of medical diagnoses and treatment procedures. However, integrating telemedicine into care requires juggling international medical culture and local cultural variations in medical delivery. Medical-governance strategies need to embed evidence-based medicine and telemedicine into different organizational set-ups. Incremental “juggling” is considered useful for solving organizational challenges.

Regarding internet and global business governance, the authors of the two reviewed papers suggest that “Focusing on the public good can be a first and crucial step to ensure an accurate, reliable, and evidence-based online presence for health for this generation and the next” [23]. They recommend that the “Internet community needs to be vigilant to ensure the reliability and trustworthiness of health information online, and take immediate action to secure the future integrity and proper governance of this important namespace for the health internet” [22]. The authors hope to encourage ICANN to appoint the World Health Organization and a multitude of governmental and health nongovernment organizations as sponsors. This presupposes that the World Health Organization has executive governance power with the ability to overrule the global internet market and the political governance within each member state. They express a need for political governance to dominate global business strategies.

The papers propose solutions for “future e-Health governance approaches to ensure the appropriate management of health could be accomplished by requesting ICANN to recategorize health as a sponsored gTLD and proactively appoint WHO as its sponsors” [23]. They suggest that the World Health Organization should develop policies to ensure accountability and transparency in gTLD operations that meet the best interests of the global health community and enforce eligibility rules regarding all future health registrants.

They propose that the World Health Organization’s possible appointment as a gTLD sponsor should be governed by a diverse, globally representative board of health stakeholders in partnership with responsible internet service providers. “This governance mechanism can have representation and be organized into subject-specific advisory panels to review and recommend content to be included on for health” [23].

In the self-governance category, consciousness and data activism is important, which resonates with an argument from Alan Peterson, who calls for “algorithmic accountability’ which highlights the potential for citizens to create alternative futures—ones oriented to fulfilling human needs rather than techno-utopian visions” [31].

### Limitations of the Review

By limiting our inclusion criteria to 2010 onwards, we might have missed important papers that could enhance the review. Qualitative reviews are not all-embracing, and the results are colored by the search criteria. Nevertheless, our paper provides valuable insights into various governance strategies at play in the realm of eHealth and valuable suggestions for further work to improve governance and scientific knowledge.

### Postscript – Governance and the Coronavirus Pandemic

The current coronavirus pandemic might provide an excellent illustration of our findings and main conclusions.

To govern and control the pandemic, collective, transparent efforts are in demand: political decisions followed by financial incitements, firm global medical knowledge and advice, international business actors’ financial and ethical decisions, and personal decisions by citizens to adhere to the advice. Following a process when the collectively negotiated goal to fight the pandemic is at stake, puts strong demands on each of these “nodes” in dynamic co-governance.

### Conclusions

We have identified 4 different governance models linked to national eHealth programs, national health ICT infrastructures, regional and local professional networks, Health Internet businesses, consumer-driven self-management solutions, and virtual health services beyond national health services.

The political model depicts governance through various management strategies to influence wider health political goals and innovation. The model also enacts eHealth governance by establishing financing schemes, infrastructures, standards, and laws. Stronger leadership, by involving stakeholders in national health IT boards, is suggested to achieve goals. The medical-governance model enacts governance within and by eHealth communities grounded in evidence and trust. The authors of papers addressing this model argue that evidence-based medicine should be the basis for the development of political-governance strategies. The internet and global business model put international business actors in dominant roles in eHealth governance, challenges the jurisdictions of nation-states, and limits the influence of international health actors, such as the World Health Organization. The authors of papers that address this model envision a dystopian future, especially for the governance of health data and services on the internet, and they argue for action by global health authorities. In the self-governance model, governance is characterized by shifting, dynamic tensions: governance and control of the individual by their submission to eHealth versus self-governance within eHealth by empowered, conscious citizens.

On the basis of this review, we conclude that to achieve health policy goals in large-scale eHealth policy programs, collective negotiations between nation-states, global policy actors, medical and self-governance actors, and global business and industry actors are essential. Digital technology affordances present opportunities for both benefit and harm concerning the realization of health policy goals, a dynamic that future studies should scrutinize. Technological affordances and both optimistic and pessimistic views deserve serious consideration.

According to our findings, further research is needed to produce knowledge about

1. How large-scale eHealth programs are realizing national and international health goals through collective negotiations
2. How the interference dilemmas between models are (creatively) accommodated and dealt with by patients or consumers to obtain quality and equal access
3. How the consequences of interference between models are (creatively) co-managed by global vendors, regional, national, and international governmental actors, and national and international nongovernment organizations to achieve health policy goals?

## Abbreviations

EHR: electronic health record
gTLDs: generic top-level domain names
HIT: health information technology
ICANN: Internet Corporation for Assigned Names and Numbers
ICT: information and communication technology
IT: information technology
NHS: National Health Services
NPfIT: National Programme for Information Technology
TLD: top-level domain
