# Correction: Elaborating Models of eHealth Governance: Qualitative Systematic Review

**DOI:** 10.2196/25853

**Published:** 2020-11-24

**Authors:** Anne Granstrom Ekeland, Line Helen Linstad

**Affiliations:** 1 Norwegian Centre for E-health Research Tromsø Norway

In “Elaborating Models of eHealth Governance: Qualitative Systematic Review” (J Med Internet Res 2020;22(10):e17214), the authors noted one error.

In the Methods section of the Abstract, the following sentence specified an incorrect date range:

We searched the PubMed database using predefined search terms and selected papers published in 2010.

This sentence has been corrected to:

We searched the PubMed database using predefined search terms and selected papers published from 2010 onwards.

The correction will appear in the online version of the paper on the JMIR Publications website on November 24, 2020, together with the publication of this correction notice. Because this was made after submission to PubMed, PubMed Central, and other full-text repositories, the corrected article has also been resubmitted to those repositories.

